# Medical Opinion and Movement

**Published:** 1910-04-23

**Authors:** 


### Medical Opinion and Movement.

### Potash in the Wassermann Reaction.

Several attempts have been made to simplify the Wassermann reaction by employing simpler reagents than those originally proposed by Wassermann. Drs. Brieger and Renz propose to substitute chlorate of potash for the hæmolytic amboceptor of Wassermann (the serum from a rabbit rendered immune against the blood corpuscles of sheep by repeated injections). In 65 cases these authors have obtained the same results with chlorate of potash as with the hæmolytic amboceptor of Wassermann. In these experiments they used a saline solution containing 1 gramme of chlorate of potash to 150 grammes of solution. The solution should be prepared fresh each time, or it is liable to undergo changes on keeping.

### Multiple Parasitism.

An extraordinary case of parasitism has been recently reported to the Paris Academy of Medicine by Drs. Raillet and Henry. A peasant woman of Normandy, aged 63, had suffered four months from marked pruritis, for which she received no treatment until her husband had the idea of collecting the crusts which she had peeled off and taking them for examination to a veterinary surgeon of a neighbouring town. This last, surprised to find a moving population in the material so collected, sent it to a doctor, and asked him to go and see the woman. The patient was found to be in an inexpressibly wretched condition of dirt and malnutrition, her whole body being covered with excoriations. Clinically, the affection somewhat resembled scabies, but no burrows could be found, and small white larvæ were noticed in the crusts. On being sent into hospital, hygienic measures and proper diet rapidly cured the patient. Microscopic examination of the crusts showed the presence of a whole fauna, the predominating genus being extremely active larvæ of the flea, though adult fleas and six different species of acarus were noticed. The only true parasites were the adult fleas and lice, which had prepared the ground for the installation and nutrition of the pseudo-parasites, including the larvæ of the fleas. The authors have only been able to find one analogous case in the literature, and that one by no means so complex as the one under discussion. Comparative pathology has, however, furnished several examples in the cat and dog. The authors end their report by expressing their surprise that it was possible for such a case to occur at the commencement of the twentieth century in one of France's richest provinces, a surprise in which we confess we share.

### Quinine in Whooping Cough.

As reported in the *Munchener Medizinische Wochenschrift*, Dr. Berliner has for some years past treated cases of whooping-cough by the introduction into the patient's nasal fossæ of an ointment consisting of 1 to 2½ parts of quinine sulphate mixed with 10 to 15 parts of vaseline, the strength varying according to the age of the patient. This ointment is introduced into the nares three times daily with a glass rod, and in order that the whole surface of the nasal canal may be reached by the drug, the patient's head is thrown well back during the introduction. The author has found that with this treatment marked improvement is noticeable in three or four days, the attacks of coughing being reduced both in number and in intensity. The drug would seem to be the more marked in its effects the younger the patient.

### Fibrin and Callus Formation.

Among other interesting papers at the recent Congress of the German Surgical Society was one by Dr. Bergel on the *rôle* played by fibrin in the formation of callus. In a previous work this author has already demonstrated that fibrin possesses the property of inducing the emigration of leucocytes and the formation of fibroblasts and connective tissue. He regards it as the cause and first condition of cicatrisation and of the formation of callus after fractures. Subperiosteal injections of fibrin in animals result in a prolific new osseous formation. Injections of serum or of red corpuscles do not produce any such result, and the author therefore concludes that the callus formation is due to the fibrin, and to no other elements of the blood. He claims, further, to have obtained very satisfactory results in cases of badly healing wounds by treating them with fibrin, and in cases of pseudarthrosis or weak callus formation by subperiosteal injections of an emulsion of fibrin in saline solution.

### Camphorated Oil in Suppurative Peritonitis.

In yet another paper Dr. Hirschel, of Heidelberg, recommended the introduction of 1 per cent. sterilised camphorated oil after operation in cases of suppurative peritonitis. Before closing the wound he pours in 100 to 300 cubic centimetres, and distributes as much as possible over the peritoneal cavity. He has treated nine serious cases in this way. Five recovered completely. The other four died of attending complications, and at the autopsy the abdominal condition was found to be quite satisfactory. The peritoneum was smooth and glistening, covered with a fine layer of oil, and without any fibrinous adhesions. In addition to producing an engorgement of the lymphatics and so preventing absorption, it is suggested that the camphorated oil envelops the bacteria and renders them innocuous. Several other surgeons spoke favourably of the effects of the treatment.

### Preparations for Abdominal Operations.

That unanimity and finality are by no means yet reached among surgeons on the subject of the proper preliminary treatment before laparotomy is evident from two papers, by Messrs. H. J. Paterson and A. J. Wallace respectively, in two recent consecutive numbers of the *Practitioner*. The former, for instance, urges that whenever possible the patient should be prepared for at least a week; that he should have only fluid food during that period, and for fifty hours preceding operation nothing at all but sterilised milk and tea. The latter advises that rarely should preparations be commenced more than thirty-six hours ahead, and more usually but twenty-four; he thinks the substitution of a purely liquid diet for one of a solid or partially solid character is unnecessary and injurious. The London surgeon orders several days' dosage with calomel, the last dose to be given two nights previous to the operation; on the morning of the day before operation he orders castor oil, and on the same evening a turpentine enema; on the morning of operation a simple soap enema is given. The Liverpool surgeon has found that an ordinary aperient twenty-four hours before operation, followed a few hours later by an enema, proves quite sufficient for all ordinary cases. Both surgeons approve highly of the acetone-iodine method of preparing the skin, and insist on careful oral hygiene. On the prevention of shock serious divergence once more becomes evident. Mr. Paterson relies mainly on "open-ether" anæsthetisation, with an injection of $\frac{1}{6}$ grain of morphia and 1/100 grain of atropine half an hour previously; Mr. Wallace is equally enthusiastic over the virtue of four or five preliminary injections of strychnine; both are fully convinced of the immense value of their respective plans, which are so diametrically opposite in kind.

### The Ætiology of Iritis.

A detailed review of five hundred cases of iritis is published in *Ophthalmology* by Drs. Jenning and Hill. First in the ætiology is syphilis, which accounted for the lesion in 307 cases, a percentage of 61.4. It may be doubted whether in private practice the proportion is quite as high as this; the authors' cases were chiefly in hospital practice. Both eyes were involved in 81 cases, one only in 226. The lesion was commonest within the first five years after infection, and, in fact, betrayed the characters of a secondary, rather than a tertiary, manifestation. Next to syphilis comes, as might be expected, rheumatism, which was responsible for 127 cases, or 25.4 per cent. It will thus be seen that these two factors together explained 86.8. Only those cases in which iritis was definitely accompanied by acute synovitis of typical rheumatic character were set down as cases of rheumatic iritis; had every case in which a history of rheumatic pains could be obtained been classified as rheumatic iritis, no doubt the percentage would have been much larger. Recurrences took place in 70 out of these 127 patients, whereas but 46 of the 307 syphilitics had more than one attack. Gonorrhœa was ascribed as the cause in 26 cases, influenza and exposure in seven each, malaria and tuberculosis in six each. Gout appears to have been found in one case only, a very striking fact probably to be explained by the comparative rarity of this disease in America; for certainly gouty iritis is more often met with than in one out of five hundred cases over here.

### Novel Method of Rhinoplasty.

A highly ingenious plan for restoring a nose which had been almost totally destroyed by the discharge of a shotgun at close quarters is described by Dr. S. H. Watts in the *Annals of Surgery*. The distal phalanx of the little finger was denuded of skin, and the nail with its matrix thoroughly removed. The tip of the bone was then wired to the patient's skull in the region of the glabella, and the arm was held in place by a plaster cast. A fortnight later the finger was amputated under local anæsthesia, and a few days later the end of the finger was trimmed off, and the proximal phalanx removed, in order to diminish the thickness of the septum and to restore the size of the nostrils. The columna was then sutured in place over the end of the finger. Then later on the skin on the dorsum of the finger was removed, and a flap of skin was turned down on the forearm and sutured to the right margin of the nasal wound; the arm was once more immobilised in plaster. After another fortnight the flap of skin was severed from the arm and its free edge stitched to the left margin of the nasal wound. The result is said to have been excellent, both cosmetically and functionally; and a series of photographs certainly supports the contention.

### Suppuration in the Accessory Sinuses.

A comprehensive research into the bacteriology of the accessory sinuses of the nose has been carried out by Drs. Logan Turner and Lewis, whose results are published in the *Edinburgh Medical Journal*. Cocci are more frequently the cause of sinus suppuration than are bacteria, and the four kinds commonly met with are pneumococci, streptococci, staphylococci, and micrococcus catarrhalis. Besides these the following are not rare: Bacillus coli, proteus and similar putrefactive bacteria, dental organisms like bac. gangrenæ pulpæ and bac. necrodentalis, bacillus influenzæ, diphtheroid bacteria, and certain obligate anaerobics. It is thought that about one-third of the cases of maxillary antral suppuration have a dental, and the rest a nasal, origin. In dealing clinically with suppuration in this chamber it is of comparatively little moment what micro-organism is present; far more important is it to commence lavage early, for if this be done within three weeks of onset an early cure is probable. This lavage should be carried out through the nose, and not through the canine fossa, a conclusion which is nowadays supported almost exclusively by those who see many of these cases. Although it is found that an old-standing streptococcus invasion is more resistant to treatment by lavage than is any other infection, yet the authors end up with the confession that there is no evidence that any special combination of organisms is responsible for the failure of treatment by lavage.

### The Abdominal Manifestations of Purpura.

An instructive article on the acute abdominal symptoms which sometimes accompany purpuric manifestations appears in *L'année Médicale de Caen*

from the pen of Dr. Paul Léger. Such symptoms are met with in many varieties of the condition. It is, however, mainly in rheumatic purpura, occurring especially in children and young adults, that abdominal symptoms are met with. This variety is characterised by a purpuric eruption, pain and swelling of the joints, and gastro-intestinal disturbances. Violent paroxysmal abdominal pain, persistent vomiting of blood-stained or even fæculent material, dysenteric blood-stained stools, or at times obstinate constipation, are the results of these last. The patient looks very ill, and the abdomen is either retracted or distended. When the purpura is so slight and transient as to escape notice, diagnosis is often a matter of great difficulty, and the condition may be mistaken for lead colic, gastric crises of tabes, poisoning, or some such surgical condition as acute peritonitis, appendicitis, intestinal obstruction, etc. Purpura of the abdominal type progresses slowly, and relapses are frequent, the abdominal pains recurring at intervals sometimes for years. Prognosis is good despite the apparent severity of the symptoms, but in some cases intussusception and perforative peritonitis have occurred. The pathogeny of the abdominal symptoms is obscure. They may be due to hæmorrhages into the intestinal wall, or to a nervous lesion of an infectious nature. Treatment is simple, and in addition to sedative drugs, such as opium and morphia, calcium chloride in large doses over a long period of time should be given. Adrenalin and fresh blood-serum may also be tried with advantage.

### Hæmorrhoids in Children.

According to Zesas, in the *Archives Générales de Chirurgie*, while hæmorrhoids are uncommon in children, they are certainly met with, and when found are due to a congenital thinness of the mucous membrane of the anorectal region, with corresponding weakness and maldevelopment of the vessel walls. As a consequence of this latter condition the venous walls are unable to support the column of blood in the hæmorrhoidal veins, and hæmorrhoids appear as bluish tumours round the anus. The condition is encouraged by any irregularity in the action of the bowels, such as constipation or diarrhœa, while abdominal tumours or enlargement of the liver may cause it. Simple ulcers of the subsphincteric region and anal excoriations play a part in the production of hæmorrhoids. Diagnosis is easy and treatment is the same as in the adult.

### Diabetes Mellitus as an Infectious Disease.

As the result of a series of experiments with the saccharomyces cerevisiæ, a fungus found in the urine of every diabetic patient, Dr. Alfred King has come to the conclusion that diabetes is a fungus disease. In a paper published in the *Monthly Cyclopædia and Medical Bulletin* he points out that these organisms are capable of forming enzymes which convert glycogen and certain foodstuffs into glucose. He has found the fungus in large quantities in the blood of 16 cases of diabetes by cultural methods. Estimation of the opsonic indices of blood to the organisms gave results which fairly represent the physical condition of the patients examined. In six cases treated with vaccines of saccharomyces cerevisiæ, the opsonic indices of the patients were brought up to the normal after three or four doses. At the same time the patients were much relieved of their symptoms, thirst becoming less, the amount of urine and the sugar output decreasing, and a feeling of well-being taking the place of the former lassitude. In treating diabetes the author recommends the use of antiseptics to remove the fungus; the employment of vaccine therapy, which causes neither local nor general disturbance, to increase the phagocytic action of the leucocytes; and the administration of suitable diet, tonics, alkalies in acidosis, deep breathing exercises to increase oxidation and the elimination of carbon dioxide; and suitable surgical and antiseptic measures in dealing with boils, carbuncles, and gangrene.

### New Technique of Spinal Anæsthesia.

Dr. Poenaru, surgeon to the Central Hospital of Craïova, calls attention, in the *Deutsche Medizinische Wochenschrift*, to some improvements he has made in the technique of spinal anæsthesia by means of stovaine. One of the disadvantages of the drug is that it decomposes, and gives a milky precipitate when in contact with an alkaline medium, and these results happen in the spinal canal as soon as the drug comes into contact with the alkaline cerebrospinal fluid. Since the alkalinity of this fluid varies in different individuals, it is impossible to know before injection how much of the drug will be precipitated and how much will enter the circulation. It is this fact which explains the variability of the results obtained from spinal injections of stovaine. The author, after experimenting with phosphoric acid, has now rejected this, and substitutes lactic acid, which he adds to the stovaine solution to inhibit precipitation. He finds that an eighth of a minim of a strong solution of lactic acid is sufficient to effect this. In addition to the acid, the author add a little adrenalin to the stovaine solution, which he finds prolongs the duration of the anæsthesia and diminishes the chances of intoxication. He employs the following technique when administering the spinal injection. To fifteen minims of a one in a thousand solution of adrenalin in a glass vessel is added 1 minim of strong lactic acid. In another glass vessel there is placed sufficient stovaine solution to amount to three-quarters of a grain of the drug. Just prior to performing spinal puncture two drops of the first solution are added to the stovaine solution, and the whole thoroughly mixed by shaking. Puncture is then performed, and 30 minims of spinal fluid withdrawn and added to the stovaine mixture, which is then well shaken again. Of this new mixture, 30 minims are drawn up into the syringe and injected into the spinal theca. The author finds that this technique ensures complete, constant, and safe anæsthesia for a period of two hours.

